# Multilevel Threshold Segmentation of Skin Lesions in Color Images Using Coronavirus Optimization Algorithm

**DOI:** 10.3390/diagnostics13182958

**Published:** 2023-09-15

**Authors:** Yousef S. Alsahafi, Doaa S. Elshora, Ehab R. Mohamed, Khalid M. Hosny

**Affiliations:** 1 Department of Information Technology, Khulis College, University of Jeddah, Jeddah 23890, Saudi Arabia; ysalsahafi@uj.edu.sa; 2 Department of Information Technology, Faculty of Computers and Informatics, Zagazig University, Zagazig 44519, Egypt; engineer_elshora@yahoo.com (D.S.E.); ehab.rushdy@gmail.com (E.R.M.)

**Keywords:** image segmentation, COVIDOA, Otsu, Kapur, Tsallis, skin cancer images

## Abstract

Skin Cancer (SC) is among the most hazardous due to its high mortality rate. Therefore, early detection of this disease would be very helpful in the treatment process. Multilevel Thresholding (MLT) is widely used for extracting regions of interest from medical images. Therefore, this paper utilizes the recent Coronavirus Disease Optimization Algorithm (COVIDOA) to address the MLT issue of SC images utilizing the hybridization of Otsu, Kapur, and Tsallis as fitness functions. Various SC images are utilized to validate the performance of the proposed algorithm. The proposed algorithm is compared to the following five meta-heuristic algorithms: Arithmetic Optimization Algorithm (AOA), Sine Cosine Algorithm (SCA), Reptile Search Algorithm (RSA), Flower Pollination Algorithm (FPA), Seagull Optimization Algorithm (SOA), and Artificial Gorilla Troops Optimizer (GTO) to prove its superiority. The performance of all algorithms is evaluated using a variety of measures, such as Mean Square Error (MSE), Peak Signal-To-Noise Ratio (PSNR), Feature Similarity Index Metric (FSIM), and Normalized Correlation Coefficient (NCC). The results of the experiments prove that the proposed algorithm surpasses several competing algorithms in terms of MSE, PSNR, FSIM, and NCC segmentation metrics and successfully solves the segmentation issue.

## 1. Introduction

Nowadays, SC is a serious illness that may afflict anyone regardless of race, gender, and age. The skin tissues’ aberrant growth is usually caused by exposure to Ultraviolet Radiation (UVR) from the Sun or tanning beds. The significance of SC lies in its potential to spread to other parts of the body if not detected and treated early [1]. According to the World Health Organization (WHO), in 2022, UVR caused over 1.5 million cases of SC. In 2020, there were 66,000 deaths from malignant melanoma and other SCs. In the United States, there are an estimated 1.1 million annual cases of SC. Melanoma, basal cell carcinoma, and squamous cell carcinoma are the three most frequent kinds of SC. Melanoma is the deadliest form of cancer [2].

Malignant melanoma can also be less deadly and more treatable if found early. It might be diagnosed in its early stages, preventing the need for an expensive treatment that would cost millions of dollars. However, detecting and accurately segmenting SC lesions pose significant challenges. One major challenge is the similarity between benign and malignant lesions in appearance, which makes it difficult for healthcare professionals to differentiate between them based on visual examination alone. Another challenge is the variability in different individuals’ lesion size, shape, color, and texture. This variability makes it challenging to develop a universal algorithm or model for accurate detection and segmentation across diverse populations.

Furthermore, detecting skin cancer requires expertise and experience from dermatologists or trained healthcare professionals. The shortage of dermatologists in many regions can lead to delays in diagnosis and treatment. Researchers are exploring various Computer-Aided Diagnostic (CAD) systems that utilize Artificial Intelligence (AI) techniques, such as machine learning and deep learning algorithms, to address these challenges. These systems aim to improve the accuracy and efficiency of skin cancer detection by analyzing large datasets of images and identifying patterns indicative of malignancy. Additionally, advancements in imaging technologies like dermoscopy have improved visualization capabilities for clinicians. Dermoscopy allows for magnified examination of skin lesions using specialized equipment that enhances surface details and structures not visible to the naked eye [3]. Image segmentation techniques first define the lesion’s borders to identify skin cancer. Image segmentation also refers to extracting interesting objects from images and analyzing their behavior to reveal the presence of a problem or sickness [4]. According to the literature, image segmentation techniques include edge detection [5], clustering [6], and thresholding-based segmentation [7].

Edge detection algorithms can identify the boundaries of skin lesions by detecting abrupt changes in pixel intensity. This technique is useful for identifying irregularities in the shape and texture of skin lesions, which are important features for diagnosing skin cancer. Edge detection can help differentiate between healthy skin and potentially cancerous regions.

Clustering techniques group pixels based on their similarity in color or intensity values. In the context of skin cancer detection, clustering algorithms can identify regions with similar color characteristics as potential lesions.

Thresholding is the most common segmentation approach due to its ease of use, simplicity, fast computation, and robustness against noise. Thresholding methods often have mechanisms to handle noisy data points [8]. The limitations of this technique include sensitivity to threshold selection: The choice of threshold(s) can significantly impact the segmentation results and difficulty with complex textures or lighting variations. Thresholding may struggle with complex textures or when the lighting conditions vary across the image.

Despite the significance of image segmentation in identifying objects of interest from medical images, some issues, such as noise contamination and artifacts from image capture, cause mistakes in the segmentation of medical images. Various smoothing approaches (for instance, developing an algorithm or tuning a filter) can decrease errors or eliminate noise. Without this step, the exact segmentation of the image may not be easy [9]. Most currently used segmentation methods depend greatly on several pre-processing methods to avert the consequences of unwanted artifacts that could impair accurate skin lesion segmentation [10].

Thresholding-based segmentation is split into two classes depending on how many thresholds were utilized to segment the image: Bilevel and multilevel [11]. A threshold value divides the image into homogenous foreground and background portions in the first class. On the other hand, multilevel splits the image using a histogram of pixel intensities into more than two portions. Since bilevel thresholding separates an image into two sections, it cannot accurately recognize images with numerous objects on colorful backgrounds. MLT is more suitable in these cases [12]. The essential step in the thresholding process is determining the optimal threshold values that effectively define the image segments.

As a result, it is defined as an optimization issue that may be addressed by parametric or nonparametric techniques [13]. In the parametric technique, the probability density function calculates parameters for every region to determine the optimal threshold values. Through this, the nonparametric technique aims to maximize a function like fuzzy entropy [14], Kapur’s entropy (maximizing class entropy) [15], and Otsu function (maximizing between-variance) [13]. Regrettably, by those techniques, determining the optimal threshold values for MLT is difficult and enormously raises the computational cost, especially as the threshold levels increase. Therefore, an efficient new alternative was necessary because of the substantial success of the meta-heuristic algorithms in numerous domains, such as communications, engineering, social sciences, transportation, and business. Researchers have focused on them to solve the challenges of MLT image segmentation [16,17,18,19,20,21].

Compared to a gray-level image, a color image depicts a scene in the real world more accurately. In image processing, different color spaces represent and analyze images. Each color space has advantages and disadvantages, making them suitable for specific applications. One commonly used color space is the Red, Green, Blue (RGB) color space. It represents colors by combining different intensities of red, green, and blue channels. RGB is widely used in digital imaging systems as it closely matches how humans perceive colors. However, RGB has limitations regarding image analysis tasks such as object detection or segmentation since it does not separate color information from brightness. Another popular color space is the Hue, Saturation, Value (HSV) color space. HSV separates the hue (color), saturation (intensity of color), and value (brightness) components of an image. This separation makes manipulating specific aspects of an image easier without affecting others. For example, changing only the hue component can alter the perceived color without changing brightness or intensity. HSV is often used in applications like image editing or tracking objects based on their color. Cyan, Magenta, Yellow, Key/Black (CMYK) is primarily used in printing processes where colors are represented using subtractive rather than additive mixing like RGB. It plays a vital role in the graphic design and printing industries. RGB is commonly defined and most gray-level segmentation techniques may be applied directly to each component of an RGB image; nonetheless, few studies [22,23,24] address how to apply MLT techniques to a color image. Borjigin et al. [22] concentrate on the RGB color space, which is the most commonly used to segment images.

The following summarizes the key contributions of this paper:

- COVIDOA is shown to deal with MLT in image segmentation.
- The hybridization of Otsu, Kapur, and Tsallis as a fitness function was used to present a skin cancer segmentation technique.
- Various segmentation levels are employed to assess the proposed technique’s performance.
- The proposed technique is compared to numerous popular meta-heuristics techniques.
- The effectiveness of the segmentation technique is validated by utilizing the MSE, PSNR, FSIM, and NCC matrices.
- The proposed technique may be expanded to accommodate various medical imaging diagnoses and used for additional benchmark images.

The next sections of this study are arranged as follows: Section 2 shows the related work. Section 3 presents the materials and methods. Section 4 describes the COVIDOA with the proposed fitness function for MLT segmentation. Section 5 shows the results and discussion. Section 6 provides conclusions and future work.

## 2. Literature Review

The most common meta-heuristic methods for dealing with the thresholding issue are the Particle Swarm Optimization (PSO) algorithm [25], Whale Optimization Algorithm (WOA) [26], Cuckoo Search Algorithm (CSA) [27], Harris Hawks Optimization Algorithm (HHOA) [28], Gray Wolf Optimization Algorithm (GWOA) [29], and Equilibrium Optimization Algorithm (EOA) [30]. In addition to these traditional methods, several newly adopted meta-heuristic methods include Chimp Optimization Algorithm (ChOA) [31], Manta Ray Foraging Optimization Algorithm (MRFOA) [32], Slime Mould Algorithm (SMA) [33], Marine Predators Algorithm (MPA) [34], Black Widow Optimization Algorithm (BWOA) [35], and artificial Gorilla Troops Optimizer (GTO) [16]. Khalid AM et al. [36] introduced a recently developed algorithm (COVIDOA). It exceeded conventional and contemporary rivals regarding the effectiveness of results.

The algorithms mentioned above have been tested on grayscale and color images. This study aims to advance the field of color image segmentation by giving an improved fitness function for COVIDOA. We believe this is the first use of the COVIDOA for image segmentation in color skin lesions images.

Numerous applications of meta-heuristics have been found. As a result, the papers that follow offer some important recent works. Rai et al. [37] evaluated all nature-inspired optimization techniques and the importance of such algorithms for MLT segmentation of images from 2019 until 2021. Sharma et al. [38] found that Kapur, Tsallis, and fuzzy entropy objective functions provided an efficient opposition-based modified firefly method for MT image segmentation. In [39], an upgraded GWO known as the Multistage Grey Wolf Optimizer (MGWO) is shown for MLT image segmentation. The proposed technique achieved superior outcomes compared to other examined approaches. In [40], a novel proposal that combines the WOA with the Virus Colony Search (VCS) Optimizer (VSCWOA) is given. The VSCWOA’s effectiveness in overcoming image segmentation issues has been proven. The proposed algorithm has been demonstrated to be very successful. In [41], a neural network-based method for segmenting medical images has been presented. The authors of [42] have proposed an improved method for ant colony optimization. The segmentation outcomes provided by the proposed method are more reliable and superior when compared to other methods. The authors of [24] used an adaptive WOA and a prominent color component for MLT of color images. A combination of lion and cat swarm optimization techniques offered the best threshold value for efficient MLT image segmentation [43]. Bhavani and Champa [44] presented a hybrid MPA and Salp Swarm Algorithm (SSA) to achieve optimal MLT image segmentation. Using an updated Firefly Algorithm (FA) with Kapur’s, Tsallis, and fuzzy entropy, an MLT image segmentation technique was given in [45]. In the EO algorithm, an Opposition-Based Learning (OBL) mechanism and the Laplace distribution were used [46] to create a modified EO method for segmenting grayscale images utilizing MLT. In [47], an MLT image segmentation technique depending on the moth swarm algorithm was suggested. The image segmentation findings demonstrate that their proposed technique outperforms the other analyzed algorithms regarding efficiency. Also, in [48], an improved Artificial Bee Colony (ABC) algorithm-based image segmentation using an MLT technique for color images has been suggested. Dynamic Cauchy mutation and OBL enhanced the elephant herding optimization method [49]. The WOA was presented in [50] to solve the image segmentation problem using Kapur’s entropy technique. The authors of [51] proposed a new MLT image segmentation technique depending on the Krill Herd Optimization (KHO) algorithm. Kapur’s entropy is used as a fitness function that needs to be maximized to reach the optimum threshold values. Furthermore, a new meta-heuristic algorithm, galactic swarm optimization, has been adapted to tackle image segmentation [52]. Anitha et al. [53] introduced a modified WOA to maximize Otsu’s and Kapur’s objective functions to enhance the threshold selection for MLT of color images. This proposed method surpassed various techniques, such as CS and PSO. In [54], RSA-SSA is a new nature-inspired meta-heuristic optimizer for image segmentation employing grayscale MLT based on RSA merged with the SSA. The authors of [55] developed an improved SSA that combines iterative mapping and a local escaping operator. This method utilizes Two-Dimensional (2D) Kapur’s entropy as the objective function and uses a nonlocal means 2D histogram to indicate the image information. A Deep Belief Network (DBN), depending on an enhanced meta-heuristic algorithm known as the Modified Electromagnetic Field Optimization Algorithm (MEFOA), was presented in [56] for analyzing SC. In [57], an improved RSA for overall optimization and choosing ideal threshold values for MLT image segmentation was used. The authors of [58] showed an innovative approach for skin cancer diagnosis according to meta-heuristics and deep learning. The Multi-Agent Fuzzy Buzzard Algorithm (MAFBUZO) combines local search agents in multiagent systems with the BUZO algorithm’s global search ability. During optimization, a suitable balance of exploitation and exploration steps is enabled. In [59], a new meta-heuristic algorithm for 2D and 3D medical gray image segmentation is proposed based on COVIDOA merged with the HHOA to benefit from both algorithms’ strengths and overcome their limitations. The COVIDOA is also used in [60] to solve the segmentation problems of satellite images.

## 3. Materials and Methods

This section presents the required materials and methods to develop the proposed technique. The multilevel thresholding is explained. The objective functions utilized in this research are also shown.

### 3.1. Multilevel Thresholding

Image thresholding transforms the grayscale or color image into a binary image, applying a threshold value to the image’s pixel intensity [61]. Pixels below that threshold convert into black, and those above it turn white. There are two classes of image thresholding: Bilevel and multilevel. Bilevel uses a single threshold value (th) to assign each pixel *P* of the image to one of two regions (*R*_1_ and *R*_2_) as stated below:

(1) $$P\in R_{1}if0\leq P<th\;P\in R_{2}ifth\leq P<L-1$$

where L represents the maximal intensity level.

Multilevel, on the other hand, divides an image into numerous separate areas by employing a variety of threshold values, as seen below:

(2) $$P\in R_{1}if0\leq P<{th}_{1},\;P\in R_{2}if{th}_{1}\leq P<{th}_{2},\;P\in R_{i}if{th}_{i}\leq P<{th}_{i+1},\;P\in R_{k}if{th}_{k-1}\leq P<{th}_{L-1},$$

where $\left\{{th}_{1},\right.$ ${th}_{2},$ …, $\left.{th}_{k-1}\right\}$ indicates various threshold values.

Maximizing a fitness function may determine the optimal values for thresholds. The three common thresholding segmentation techniques are Otsu’s, Kapur’s, and Tsallis’s. Every technique suggests a distinct fitness function that must be maximized to find the ideal threshold values. The three techniques are explained in the next subsections briefly. Additionally, red, green, and blue are the three main color components in an RGB image, so these thresholding techniques are used three times to obtain the best threshold values for each of the three colors.

#### 3.1.1. Otsu’s (Between-Class Variance) Method

This method is a variance-based technique suggested in [13] to find the optimal threshold values separating the heterogeneity of an image by maximizing the between-class variance. It is referred to as a nonparametric segmentation technique that splits the pixels of the grayscale or color image into various areas based on the pixel intensity values [62].

Let us suppose that we have L as the grayscale image’s intensity level or each color image’s channel with N pixels, and the number of pixels with gray level i is calculated by $x_{i}$. The gray level’s probability is given as:

(3) $$P_{i}^{C}=\frac{x_{i}^{C}}{N},\;P_{i}^{C}\geq 0,\sum_{i=1}^{L}P_{i}^{C}=1$$

Bilevel thresholding divides the original image, and the between-class variance of two categories is determined as:

(4) $${\;x}^{C}\left(t\right)=\sigma_{0}^{C}+\sigma_{1}^{C}$$

(5) $${\;\sigma }_{0}^{C}=\omega_{0}^{C}{\left(\mu_{0}^{C}-\mu_{T}^{C}\right)}^{2}$$

(6) $${\;\sigma }_{1}^{C}=\omega_{1}^{C}{\left(\mu_{1}^{C}-\mu_{T}^{C}\right)}^{2}$$

(7) $${\;\mu }_{T}^{C}=\sum_{i=0}^{G-1}iP_{i}^{C}$$

The average level of bilevel classes is shown as follows:

(8) $$\mu_{0}^{C}=\sum_{i=0}^{G-1}\frac{iP_{i}^{C}}{\omega_{0}^{C}}$$

(9) $${\;\mu }_{1}^{C}=\sum_{i=1}^{G-1}\frac{iP_{i}^{C}}{\omega_{1}^{C}}$$

The following is a representation of the classes’ cumulative probability:

(10) $$\omega_{0}^{C}=\sum_{i=0}^{m-1}P_{i}^{C}$$

(11) $$\omega_{1}^{C}=\sum_{i=m}^{G-1}P_{i}^{C}$$

Consequently, the optimal threshold $t^{*C}$ of Otsu is calculated by maximizing the class variance as:

(12) $$t^{*C}=\arg max\left(\sigma_{0}^{C}+\sigma_{1}^{C}\right)$$

The image is categorized into f classes and with f−1 threshold values. The Otsu between-class variance is shown as:

(13) $$x^{C}\left(t\right)\;=\sum_{i=0}^{y-1}\sigma_{i}^{C}$$

The optimal thresholding values $\left(t_{1}^{*C},\;t_{2}^{*C},\;\ldots t_{y-1}^{*C}\right)$ are determined by maximizing $\sigma_{B}^{C}$ as follows:

(14) $$\left(t_{1}^{*C},t_{2}^{*C},\ldots t_{f-1}^{*C}\right)=\arg\;\left\{\sigma_{B}^{C}\right.\left.\left(t_{1}^{*C},t_{2}^{*C},\ldots t_{f-1}^{*C}\right)\right\},\;0\leq t_{1}^{*comp}\leq ,...t_{y-1}^{*comp}\leq L-1$$

(15) $$\sigma_{B}^{C}=\sigma_{0}^{C}+\sigma_{1}^{C}+\ldots +\sigma_{y-1}^{C}$$

(16) $$\sigma_{0}^{C}=\omega_{0}^{C}{\left(\mu_{0}^{C}-\mu_{T}^{C}\right)}^{2},\;\sigma_{1}^{C}=\omega_{1}^{C}{\left(\mu_{1}^{C}-\mu_{T}^{C}\right)}^{2},\;\sigma_{f-1}^{C}=\omega_{f-1}^{C}{\left(\mu_{f-1}^{C}-\mu_{T}^{C}\right)}^{2}$$

The following are the average levels of f classes:

(17) $$\mu_{0}^{C}=\sum_{i=0}^{t_{1}-1}\frac{iP_{i}^{C}}{\omega_{0}^{C}}$$

(18) $$\mu_{1}^{C}=\sum_{i=m_{1}}^{t_{2}-1}\frac{iP_{i}^{C}}{\omega_{1}^{C}}$$

(19) $$\mu_{f-1}^{C}=\sum_{i=t_{f-1}}^{L-1}\frac{iP_{i}^{C}}{\omega_{f-1}^{C}}$$

Similarly, when applying Otsu’s method, *C* = 1, 2, 3, where *C* stands for the RGB image channels and *C* = 1 represents the grayscale image.

#### 3.1.2. Kapur’s Entropy (Maximum Entropy Method)

Another unsupervised automated thresholding approach is Kapur’s method, which chooses the optimal thresholds depending on the entropy of split classes [15]. The entropy is employed by computing the probability distribution of the gray-level histogram [63] to predict information from an image. The objective function for Kapur’s maximization in bilevel thresholding is as follows:

(20) $$x\left(t\right)=K_{0}^{C}+K_{1}^{C}$$

where

(21) $$K_{0}^{C}=-\sum_{i=0}^{t-1}\frac{P_{i}^{C}}{\omega_{0}^{C}}ln\frac{P_{i}^{C}}{\omega_{0}^{C}}$$

(22) $${\;\omega }_{0}^{C}=\sum_{i=0}^{t-1}P_{i}^{C}$$

(23) $$K_{1}^{C}=-\sum_{i=0}^{L-1}\frac{P_{i}^{C}}{\omega_{1}^{C}}ln\frac{P_{i}^{C}}{\omega_{1}^{C}}$$

(24) $${\;\omega }_{1}^{C}=\sum_{i=0}^{G-1}P_{i}^{C}$$

(25) $$C=\left\{\begin{array}{c} 1,\;2,\;3\;if\;image\;is\;RGB\\ 1,\;if\;image\;gray\;scale \end{array}\right.$$

where

(26) $$P_{i}^{C}=\frac{h_{i}^{C}}{\sum_{i=0}^{G-1}h^{C}\left(i\right)}$$

The optimum threshold value is as follows:

(27) $$t_{1}^{*C}\left(t\right)=\arg max\left({K\;}_{0}^{C}+{\;K}_{1}^{C}\right)$$

Kapur’s multilevel thresholding extension is shown as follows:

(28) $$x\left(t\right)=\sum_{i=0}^{y-1}H_{i}^{C}$$

The image is split into f classes by f−1 thresholding values. Extension of Kapur’s entropy for multilevel thresholding image segmentation is stated as:

(29) $$K_{0}^{C}=-\sum_{i=0}^{t_{1}-1}\frac{P_{i}^{C}}{\omega_{0}^{C}}ln\frac{P_{i}^{C}}{\omega_{0}^{C}}$$

(30) $$\omega_{0}^{C}=\sum_{i=0}^{n-1}P_{i}^{C}$$

(31) $$K_{1}^{C}=-\sum_{i=t_{1}}^{n-1}\frac{P_{i}^{C}}{\omega_{1}^{C}}ln\frac{P_{i}^{C}}{\omega_{1}^{C}}$$

(32) $$\omega_{1}^{C}=\sum_{i=t_{1}}^{t_{2}-1}P_{i}^{C}$$

(33) $$K_{j}^{C}=-\sum_{i=t_{j}}^{t_{j+1}-1}\frac{P_{i}^{C}}{\omega_{j}^{C}}ln\frac{P_{i}^{C}}{\omega_{j}^{C}}$$

(34) $${\;\omega }_{j}^{C}=\sum_{i=t_{j}}^{t_{j+1}-1}P_{i}^{C}$$

(35) $$K_{f-1}^{C}=-\sum_{t_{f-1}}^{G-1}\frac{P_{i}^{C}}{\omega_{f-1}^{C}}ln\frac{P_{i}^{C}}{\omega_{f-1}^{C}}$$

(36) $${\;\omega }_{f-1}^{C}=\sum_{i=t_{f-1}}^{G-1}P_{i}^{C}$$

The optimal multilevel thresholding in multidimensional optimization issues is utilized to calculate f−1 optimum threshold values, $t_{1\;}$*,* $t_{2\;}$,…, $t_{f-1\;}$. Consequently, the objective function of Kapur’s entropy is presented as follows:

(37) $$\left(t_{1}^{*C},t_{2}^{*C},\ldots t_{f-1}^{*C}\right)=\arg\;max\left(\sum_{i=0}^{y-1}K_{i}^{C}\right)$$

#### 3.1.3. T’sallis Entropy Method

T’sallis entropy is also called nonextensive entropy. It has the benefit of using the global and objective properties of the images [64]. Depending on the multifractal theory, Tsallis entropy can be represented using a common entropic formula:

(38) $$S_{q}=\frac{1-\sum_{i=1}^{k}{\left(P_{i}\right)}^{q}}{q-1}$$

where *k* denotes the image’s total number of possibilities and *q* is the T’sallis parameter or entropic index.

T’sallis entropy can be characterized by a pseudo additively entropic rule based on Equation (39):

(39) $$S_{q}\left(A^{C}+B^{C}\right)=S_{q}\left(A^{C}\right)+S_{q}\left(B^{C}\right)+\left(1-q\right)\cdot S_{q}\left(A^{C}\right)\cdot S_{q}\left(B^{C}\right)$$

(40) $$C=\left\{\begin{array}{c} 1,2,\;3\;if\;RGB\;image\;\\ 1\;if\;Gray\;Scale\;image\; \end{array}\right.$$

Assume that $\left\{1,2,\ldots ,\right.\left.G\right\}$ represents the image gray levels and {$P_{i}=$ $P_{1},\;\left.{P_{2},\ldots ,P}_{G}\right\}$ is the gray intensity points’ probability distribution. Two classes, *A* and *B*, may be created for the background and the object of interest, respectively, followed by the supplied Equation (41).

(41) $$P_{A}^{C}=\frac{P_{1}^{C}}{P^{CA}},\;\frac{P_{2}^{C}}{P^{CA}},\ldots ,\frac{P_{t}^{C}}{P^{CA}},\;\mathrm{and}\;P_{B}^{C}=\frac{P_{t+1}^{C}}{P^{CB}},\;\frac{P_{t+2}^{C}}{P^{CB}},\ldots ,\frac{P_{G}^{C}}{P^{CB}}$$

where $P^{CA}$ *=* $\sum_{i=1}^{t}P_{i}^{C}$ and $P^{CB}$ *=* $\sum_{i=t+1}^{G}P_{i}^{C}$.

Tsallis entropy can be classified as the following for each class:

(42) $$S_{q}^{CA}\left(t\right)=\;\frac{1-\sum_{i=1}^{t}{\left(P_{i}^{C}/P^{CA}\right)}^{q}}{q-1},\;S_{q}^{CB}\left(t\right)=\frac{1-\sum_{i=t+1}^{G}{\left(P_{i}^{C}/P^{CB}\right)}^{q}}{q-1}$$

The optimum threshold value for bilevel thresholding may be obtained by using the objective function with minimal computational effort for the gray level for which this occurs:

(43) $$T_{opt}=argmax\left[S_{q}^{CA}\left(t\right)+S_{q}^{CB}\left(t\right)+\left(1-q\right)\cdot S_{q}^{CA}\left(t\right)\cdot S_{q}^{CB}\left(t\right)\right]$$

Subject to the enumerated restriction:

(44) $$\left|P^{CA}+P^{CB}\right|-1<S<1-\left|P^{CA}+P^{CB}\right|\;where\;S\left(t\right)=\left[S_{q}^{CA}\left(t\right)+S_{q}^{CB}\left(t\right)+\left(1-q\right)\cdot S_{q}^{CA}\left(t\right)\cdot S_{q}^{CB}\left(t\right)\right]$$

The formulation mentioned above may easily be expanded for multilevel thresholding utilizing Equation (45).

(45) $$\left[T_{1},T_{2},\ldots ,T_{m}\right]=\arg max\left[\begin{array}{c} S_{q}^{C1}\left(t\right)+S_{q}^{C2}\left(t\right)+\ldots +S_{q}^{CM}\left(t\right)+\\ \left(1-q\right)\cdot S_{q}^{C1}\left(t\right)\cdot S_{q}^{C2}\left(t\right)\ldots S_{q}^{CM}\left(t\right) \end{array}\right]$$

where

(46) $$\begin{array}{c} S_{q}^{C1}\left(t\right)=\frac{1-\sum_{i=1}^{t1}{\left(P_{i}^{C}/P^{C1}\right)}^{q}}{q-1},\;S_{q}^{C2}\left(t\right)=\frac{1-\sum_{i=t1+1}^{t2}{\left(P_{i}^{C}/P^{C2}\right)}^{q}}{q-1},\;\mathrm{and}\\ S_{q}^{CM}\left(t\right)\;=\frac{1-\sum_{i=t_{m+}1}^{G}{\left(P_{i}^{C}/P^{CM}\right)}^{q}}{q-1},\;M=m+1 \end{array}$$

Subject to the enumerated restriction:

(47) $$\left|P^{C1}+P^{C2}\right|-1<S^{C1}<1-\left|P^{C1}+P^{C2}\right|,\left|P^{C2}+P^{C3}\right|-1<S^{C2}\;<1-\left|P^{C2}+P^{C3}\right|,\;\mathrm{and}\;\left|P^{C(m)}+P^{C(m+1)}\right|-1<S^{m}<1-\left|P^{C(m)}+P^{C(m+1)}\right|$$

Here, in Equation (47), $P^{C1}$,$\;P^{C2}$, …, $P^{C(m+1)}$, corresponding to $S^{C1}$,$S^{C2}$, …, $S^{CM}$, can be obtained using $T_{1},T_{2},\ldots ,T_{m}$.

#### 3.1.4. Proposed Fitness Function

A hybrid fitness function determines the fitness of COVIDOA solutions in image segmentation issues. This hybrid function is created by applying weights to the Otsu, Kapur, and Tsallis functions, as shown in Equation (48).

(48) $$F_{Hybrid}={aF}_{Otsu}+bF_{Kapur}+{cF}_{T^{\prime }sallis}$$

where *a*, *b*, and *c* $\epsilon \left[0,1\right]$ are the weights related to the three fitness functions, and *a* + *b* + *c* = 1. The suggested fitness function concurrently optimizes the Otsu, Kapur, and Tsallis methods and carries this out more accurately. We tried several different combinations of *a*, *b*, and *c* values. We found that the most effective outcomes were obtained with these values: *a* = 0.6, *b* = 0.3, and *c* = 0.1. We carried out some experiments on a collection of skin cancer color images to prove these values are the best. In Section 5, the results are displayed.

## 4. COVID Optimization Algorithm with the Proposed Fitness Function

Recently, the population-based optimization method COVIDOA was proposed to model coronavirus replication as it enters the human body [36,65].

Coronavirus replication comprises four major phases, which are listed below:

1. Virus entry and uncoating

Spike protein, one of the structural proteins of the coronavirus, is responsible for the particles’ attachment to human cells when a person becomes infected with COVID-19 [66]. When a virus enters a human cell, its contents are released.

2. Virus replication

The virus attempts to replicate itself to hijack other healthy human cells. The frameshifting approach is the virus’s method of reproduction [67]. Frameshifting is the process of shifting the reading frame of a virus’s protein sequence to another reading frame, which results in the synthesis of numerous new viral proteins, which are subsequently combined to produce new virus particles. There are several different sorts of frameshifting techniques; nonetheless, the most common is +1 frameshifting, which is the following step [68]:

▪ +1 frameshifting technique

The parent virus particle (parent solution) elements are shifted one step in the right direction. The first element is lost as a result of +1 frameshifting. The first element in the proposed algorithm is assigned a random value within the limit [*Lb*, *Ub*] in the following manner:

(49) $$S_{k}\left(1\right)\;=\mathrm{rand}\;\left(Lb,Ub\right)$$

(50) $$S_{k}\left(2:D\right)=P(1:D-1)$$

where *Lb* and *Ub* are the lower and upper limits for the variables in each solution, P represents the parent solution,$S_{k}$ is the *k*th produced viral protein, and D is the problem dimension.

3. Virus mutation

Coronaviruses exploit mutation to avoid detection by the human immune system [69]. The proposed algorithm applies the mutation on a previously formed viral particle (solution) to generate a new one in the following manner:

(51) $$Z_{i}\;=\left\{\begin{array}{c} r\;if\;rand(0,1)<MR\\ X_{i}\;otherwise\; \end{array}\right.$$

The symbol *X* denotes the solution before mutation, *Z* is the mutated solution, $X_{i}$ $and\;Z_{i}$ are the ith element in the old and new solutions, i = 1, …, *D*, *r* is a random value from the limit [*Lb*, *Ub*], and MR is the mutation rate.

4 New virion release

The newly formed virus particle exits the infected cell for more healthy cells. In the proposed algorithm, if the fitness of the new solution is greater than the fitness of the parent solution, the parent solution is replaced with the new one. Otherwise, the parent solution is still in place.

**Figure 1:**
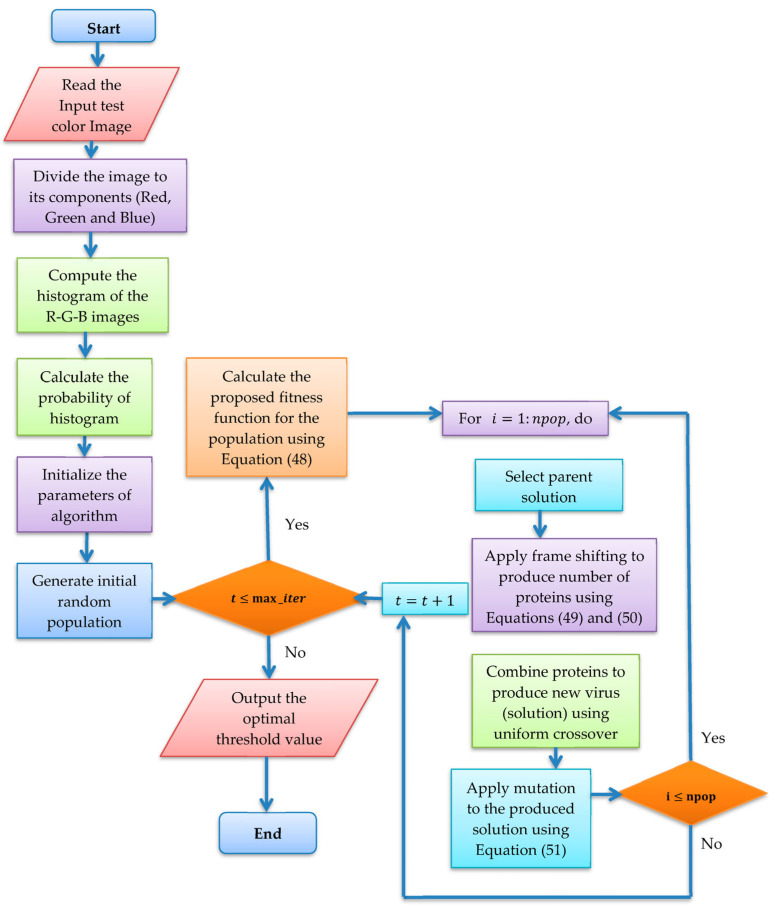
Flow chart of the COVIDOA with proposed fitness function.

The COVIDOA flow chart with the proposed fitness function for MLT segmentation of skin lesion images is depicted in Figure 1.

### Computational Complexity Analysis

According to the structure of COVIDOA, it mostly involves initialization, fitness evaluation, and updating of COVIDOA solutions. Where the number of solutions is  N, D is the dimension of the problem, and T is the maximum number of iterations. The calculation is as follows: The time complexity for initialization is  O(N). Additionally, the COVIDOA calculates the fitness of each solution with a complexity of  O(T×N×D), and the computational complexity of the update of the solution vector of all solutions is  O(N×D). Consequently, the total computational complexity of COVIDOA is  O(N×T×D).

## 5. Experimental Results and Discussion

This section begins with a summary of the datasets utilized for testing. Then, we illustrate the parameter settings for the proposed and state-of-the-art algorithms, followed by the evaluation metrics utilized to compare the outcomes. The numerical outcomes of testing the proposed algorithm and its competitors are then shown. Finally, we accomplished a comparative study of the collected outcomes.

### 5.1. Dataset

This paper uses SC images from the International Skin Imaging Collaboration (ISIC). This multinational collaboration has created the biggest public archive of dermoscopic skin images globally [70] and it is used to evaluate the proposed algorithm’s performance. More than 12,500 images across three tasks are included in this dataset.

Our experiments involve segmenting 10 color images for SC using two, three, four, and five threshold levels. Those images are selected randomly from the ISIC datasets to validate the performance of COVIDOA.

Table 1 depicts the histograms of each component and the original image, as red, green, and blue represent the three components of a color image. It is important to mention that the taken images are given new names like img1, img2, img3, img4, and so on.

### 5.2. Parameter Setting

The proposed algorithm’s MLT segmentation outcomes are evaluated using various criteria and compared to five popular meta-heuristic algorithms. These algorithms are AOA [71], SCA [72], RSA [73], FPA [74], SOA [75], and GTO [16].

These algorithms were chosen for comparison for the following reasons:

- They have demonstrated their superior capacity to solve several optimization challenges, particularly image segmentation.
- The majority of them are current and have been published in reliable sources.
- Their MATLAB implementations are freely accessible on the MATLAB website (https://matlab.mathworks.com/ accessed on 18 August 2023).

All experiments were conducted using a laptop equipped with an Intel (R) Core (TM) i7-1065G7 CPU, 8.0 GB of RAM, and the Windows 10 Ultimate 64-bit operating system. All of the algorithms were created with the MATLAB R2016b developing environment. As previously stated, all algorithms are tested across 30 independent runs with a population size of 50 and a maximum iteration count of 100 for each input SC test image. For all algorithms, the simulation setting is the same.

### 5.3. Performance Evaluation Criteria

The proposed algorithm’s performance is evaluated by four performance metrics: MSE, PSNR, FSIM, and NCC [76]. These metrics are summarized below:

#### 5.3.1. Mean Square Error (MSE)

MSE is frequently employed to calculate the difference between the segmented and original images. It is computed in the following manner:

(52) $$MSE=\frac{1}{M\times N}\sum_{i=1}^{M}\sum_{j=1}^{N}{\left[F(i,j)-f(i,j)\right]}^{2}$$

Here, $F\left(i,j\right),$ f(i,j) are the intensity level of the original and segmented image within the *i*th row and *j*th column, respectively. *M* and *N* are the image’s row and column numbers, respectively.

#### 5.3.2. Peak Signal-to-Noise Ratio (PSNR)

Another metric known as PSNR is frequently employed to quantify image quality.

It refers to the ratio of the square of the maximum gray level $255^{2}$, and the MSE between the original and separated one is computed as follows:

(53) $$PSNR=10\left(\frac{255^{2}}{MSE}\right)$$

MSE is computed using the equation mentioned above. Increasing PSNR is necessary to obtain higher quality.

#### 5.3.3. Feature Similarity Index Metric (FSIM)

FSIM is utilized to compute the structural similarity of two images in the following manner:

(54) $$FSIM\left(F,f\right)=\frac{\sum_{x\in Ω}S_{L}\left(X\right).{PC}_{m}(x)}{\sum_{x\in Ω}{PC}_{m}(x)}$$

where $S_{L\;}\left(X\right)$ indicates the resemblance between the two images, PC is the phase congruence, and Ω relates to the image’s spatial domain. The FSIM’s highest possible value, representing total similarity, is 1. A higher FSIM value enhances the thresholding process’s performance [77].

#### 5.3.4. Normalized Correlation Coefficient (NCC)

NCC is a metric for determining how closely two images are connected. NCC’s absolute value varies between 0 and 1. A value of 0 shows no relationship between the two images, and 1 denotes the most powerful possible relationship. The greater the absolute value of NCC, the stronger the association between the two images. NCC between the original image $F\left(i,j\right)$ and segmented images $f\left(i,j\right)$ is estimated in the following manner:

(55) $$NCC=\frac{\sum_{i=0}^{M-1}\sum_{j=0}^{N-1}\left[F\left(i,j\right)\times f\left(i,j\right)\right]}{\sqrt{\sum_{i=0}^{M-1}\sum_{j=0}^{N-1}[F\left(i,j\right)\times f\left(i,j\right)]\times \sum_{i=0}^{M-1}\sum_{j=0}^{N-1}\left[F\left(i,j\right)\times f\left(i,j\right)\right]}}$$

### 5.4. Experimental Results

This subsection displays the numerical outcomes of testing the COVIDOA to choose the optimal threshold values utilizing the proposed fitness function. These outcomes are evaluated against the state-of-the-art AOA, SCA, RSA, FPA, SOA, and GTO algorithms. The experiments used two, three, four, and five threshold values. We ran the COVID optimization algorithm with classic Otsu, Kapur, and T’sallis methods, and then the outcomes of these fitness functions were compared with those of using the proposed fitness function.

**Figure 2:**
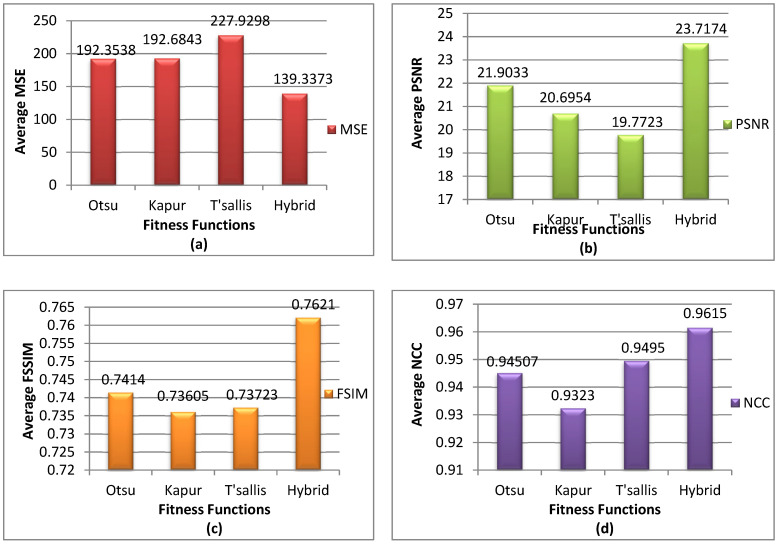
The average results of (**a**) MSE, (**b**) PSNR, (**c**) FSIM, (**d**) NCC for the COVIDOA with all fitness functions.

The outcomes are represented in Table 2, and Figure 2 depicts the average. From these results, we confirmed that the proposed fitness function surpasses all other fitness functions.

We used the proposed fitness function, as seen in Equation (48). Table 3 displays COVIDOA segmented images for all SC test images utilized in the experiments. Table 4 displays the graphs illustrating the optimal COVIDOA threshold values for RGB channels for the last test image for levels 2, 3, 4, and 5.

Table 5, Table 6, Table 7 and Table 8 provide the average findings of the corresponding MSE, PSNR, FSIM, and NCC evaluation matrices. The highest values of the thresholding approach, which produces the best results, are bolded in these tables. They show the optimal quality segmentation.

Higher mean values for PSNR, FSIM, and NCC indicate a more accurate and effective algorithm, while the lowest mean value denotes the optimum MSE value.

Table 5 lists the average values for the MSE metric. The best MSE result has the lowest mean value. It is important to note that COVIDOA surpasses all other algorithms (as previously indicated), particularly in img7 and img9, which have fewer values with all threshold levels. The SCA has lower MSE values in img1 (levels 5) and img6 (level 3), as well as the FPA in img1 (level 4), img2, and img10 (level 2).

The PSNR values are shown in Table 6 for every algorithm; a higher mean value implies superior segmentation quality. It should be noted that the COVIDOA surpasses all other algorithms in most cases.

The FSIM measure’s mean values are displayed in Table 7. This statistic examines and analyzes how well an image’s features are retained after processing. The SCA presents superior results in img1 (level 2) and img2 (level 5). Except for a few cases, the test images are not much improved by the AOA, SCA, RSA, FPA, and SOA. In comparison, the COVIDOA surpasses other algorithms in terms of FSIM on most test images.

The mean NCC values and NCC outcomes of the proposed technique (COVIDOA) outperform the other comparable algorithms, as shown in Table 8. The RSA provides a better value at only one level in img3 (level 3), GTO in img2 (levels 2 and 5), and the AOA in img2 (level 2) and img7 (level 3). The SCA gives higher results in only one image, img1 (levels 2 and 4).

**Figure 3:**
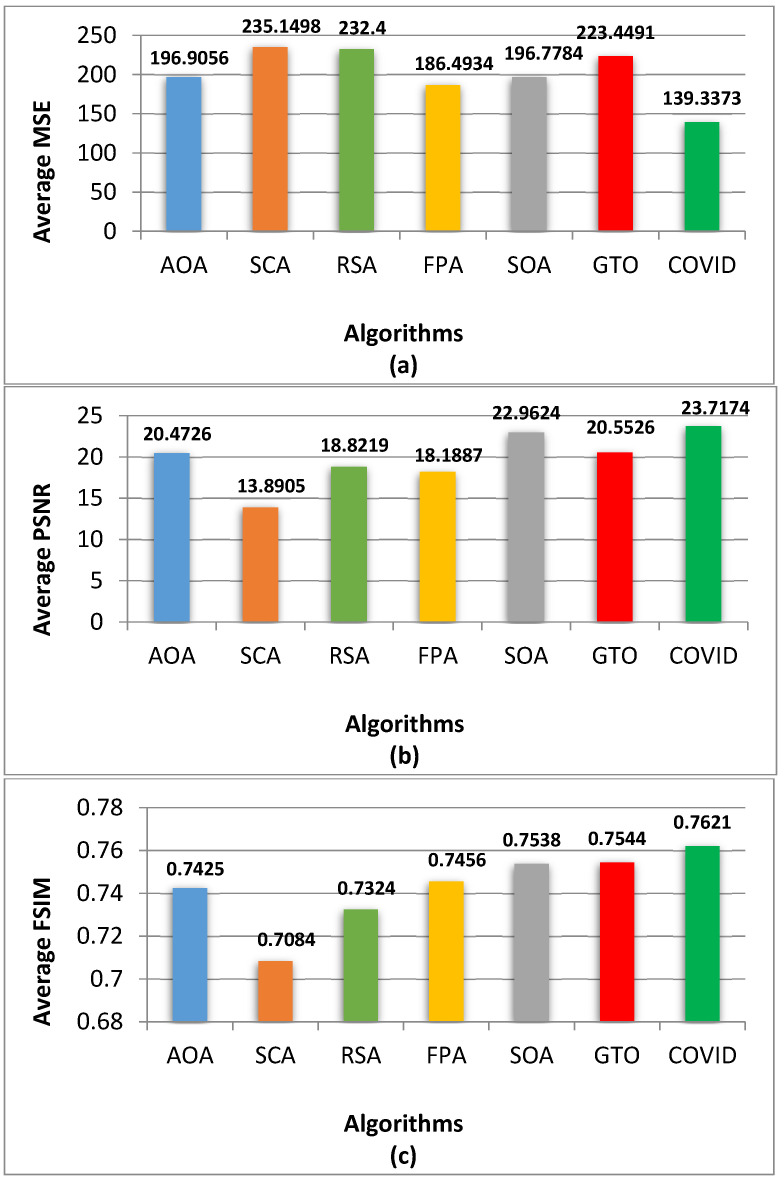

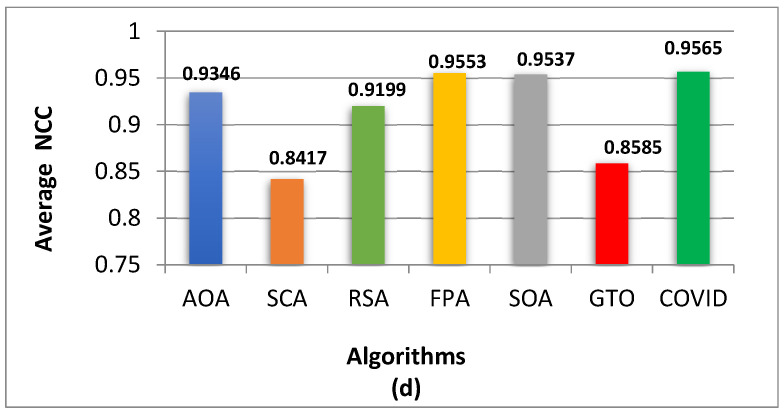
The average results of (**a**) MSE, (**b**) PSNR, (**c**) FSIM, (**d**) NCC for all algorithms.

The results of comparing the COVIDOA to other algorithms are shown in Figure 3 for the overall average values for MSE, PSNR, FSIM, and NCC.

According to Figure 3, the COVIDOA has the lowest average MSE for skin lesion images. All four measures’ bar charts indicate that the COVIDOA is superior. The highest PSNR, FSIM, and NCC values produced by COVIDOA reflect the superior caliber of the segmented images.

## 6. Conclusions and Future Work

SC is among the most prevalent kinds of cancer; consequently, early detection can significantly lower the related mortality rate. Image segmentation is essential to any CAD system for extracting regions of interest from SC images to enhance the classification phase. One of the most successful and effective techniques for segmenting images is thresholding. This work addresses the challenge of choosing the appropriate threshold value for segmenting images in MLT. The COVIDOA with the proposed fitness function was applied to a collection of color SC images. The COVIDOA’s performance is validated using 10 skin lesion images and compared to six other meta-heuristic algorithms, AOA, SCA, RSA, FPA, SOA and GTO using a range of two to five different threshold values. The performance of the proposed algorithm has been evaluated using the following metrics: MSE, PSNR, FSIM and NCC. The outcomes of the experiments proved that the proposed fitness function improves the COVIDOA with classic Otsu, Kapur, and T’sallis fitness functions for the segmentation issue. According to the results, the COVIDOA surpasses all other algorithms regarding MSE, PSNR, FSIM, and NCC segmentation measures. The proposed method may solve various image processing difficulties and improve applications, including visualization, computer vision, CAD, and image classification. Future research should widen the examined image dataset and raise the threshold values to obtain accurate results. Furthermore, the proposed method must be evaluated with other different meta-heuristic optimization and deep learning methods to enhance the outcomes of segmentation techniques.

Future studies might involve combining the innovative COVIDOA with one of the existing meta-heuristics to address the MLT problem for skin lesion segmentation in color images. The COVIDOA developed here can solve more complex, real-world optimization problems. The proposed COVIDOA’s accuracy and resiliency may be further evaluated in various engineering and real-world situations with an unknown search space.

## Figures and Tables

**Table 1:** The original SC images and their histogram of constituent colors (red, green, and blue).

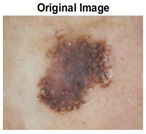	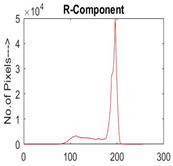	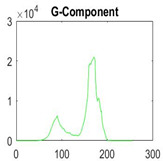	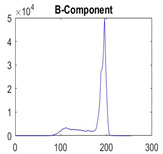
Img1	--- Pixel Intensity (0–255) --->		
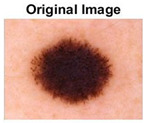	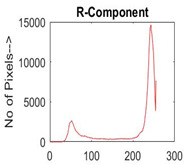	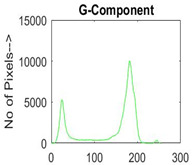	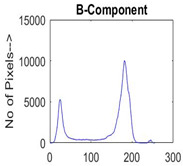
Img2	--- Pixel Intensity (0–255) --->		
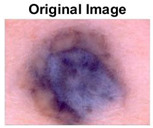	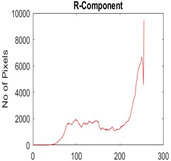	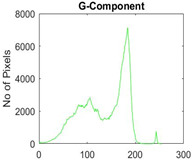	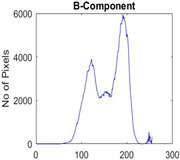
Img3	--- Pixel Intensity (0–255) --->		
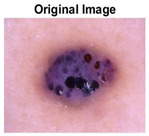	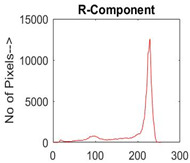	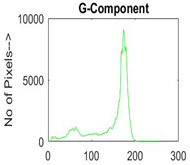	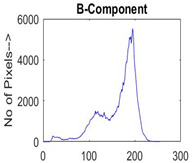
Img4	--- Pixel Intensity (0–255) --->		
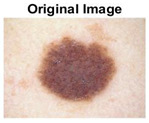	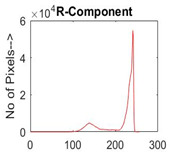	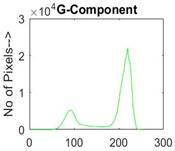	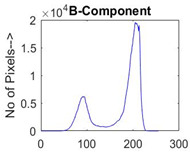
Img5	--- Pixel Intensity (0–255) --->		
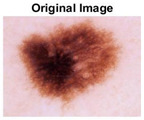	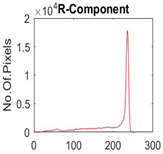	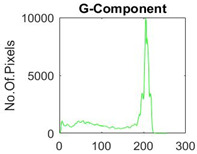	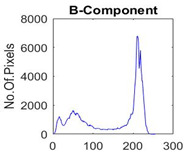
Img6	--- Pixel Intensity (0–255) --->		
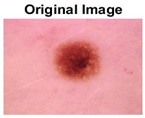	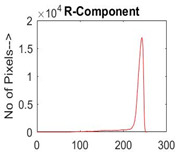	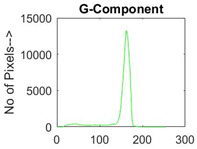	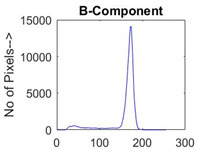
Img7	--- Pixel Intensity (0–255) --->		
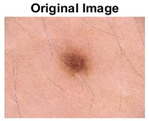	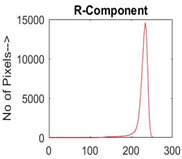	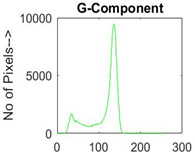	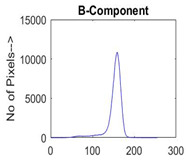
Img8	--- Pixel Intensity (0–255) --->		
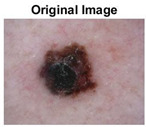	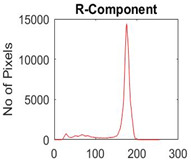	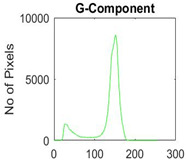	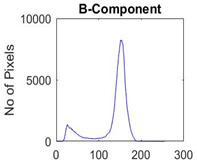
Img9	--- Pixel Intensity (0–255) --->		
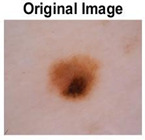	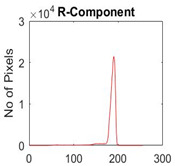	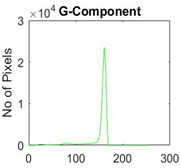	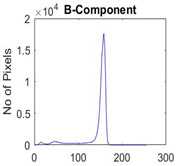
Img10	--- Pixel Intensity (0–255) --->		

**Table 2:** The results of the COVIDOA with all fitness functions.

Image	Th	MSE				PSNR				FSIM				NCC			
		Otsu	Kapur	T’sallis	Hybrid	Otsu	Kapur	T’sallis	Hybrid	Otsu	Kapur	T’sallis	Hybrid	Otsu	Kapur	T’sallis	Hybrid
Img1	2	**176.899**	229.458	231.769	244.547	13.6755	**13.8419**	12.5877	12.8173	0.7278	0.7259	0.7268	**0.7387**	**0.9463**	0.9030	0.8988	0.9152
	3	201.123	**191.807**	198.215	196.075	15.1048	15.4739	**17.6411**	16.2881	0.7543	0.7320	0.7387	**0.7654**	**0.9365**	0.9083	0.9055	0.9238
	4	**153.886**	193.375	167.093	196.123	16.5593	17.5392	20.7069	**20.9091**	0.7577	0.7528	0.7866	**0.7999**	0.9304	0.9173	0.9303	**0.9372**
	5	**136.530**	177.843	197.954	162.939	19.1578	18.9927	21.7588	**22.6851**	0.7868	0.7584	0.8109	**0.8179**	0.9371	0.9244	0.9396	**0.9530**
Img2	2	**229.326**	235.223	245.039	241.141	14.5593	14.9518	15.273	**15.3446**	0.6429	0.6352	0.6436	**0.6503**	0.9584	0.9304	0.9534	**0.9627**
	3	230.973	234.952	231.555	**222.253**	18.0646	16.2142	17.7582	**18.1292**	**0.6773**	0.6528	0.6646	0.6688	0.9732	0.9596	0.9799	**0.9808**
	4	213.243	211.400	215.130	**206.814**	19.4249	18.6225	**19.6770**	19.4283	**0.7082**	0.6696	0.7021	0.7018	0.9836	0.9772	0.9849	**0.9852**
	5	225.660	195.168	198.232	**186.758**	20.7957	19.8970	**20.2702**	20.6270	**0.7367**	0.6966	0.7180	0.7244	0.9872	0.9832	0.9862	**0.9879**
Img3	2	**229.326**	235.223	245.039	241.141	14.5593	14.9518	15.273	**15.3446**	0.6429	0.6352	0.6436	**0.6503**	0.9584	0.9304	0.9534	**0.9627**
	3	230.973	234.952	231.555	**222.253**	18.0646	16.2412	17.7582	**18.1292**	**0.6673**	0.6528	0.6646	0.6688	0.9732	0.9596	0.9799	**0.9808**
	4	213.243	211.400	215.130	206814	19.4249	18.6225	**19.6770**	19.4283	**0.7082**	0.6696	0.7021	0.7018	0.9836	0.9772	0.9849	**0.9852**
	5	172.591	187.591	117.673	**167.079**	17.7406	19.7514	21.058	**21.3038**	0.7268	0.7090	0.7370	**0.7308**	0.9444	0.9598	0.9667	**0.96804**
Img4	2	225.398	232.498	234.029	**202.004**	13.8852	12.5221	**15.8050**	14.4077	0.6709	0.6771	**0.6831**	0.6717	0.9167	0.7978	0.9205	**0.9292**
	3	202.052	224.445	211.753	**196.021**	17.4387	17.7139	18.9657	**19.5479**	0.6943	0.6893	0.7111	**0.7169**	0.9535	0.9485	0.9594	**0.9621**
	4	193.891	222.139	212.506	**187.264**	20.2741	18.8596	19.9225	**20.7163**	**0.7443**	0.7117	0.7178	0.7359	**0.9719**	0.9624	0.9676	0.9693
	5	**158.853**	183.872	171.182	21.6327	21.5364	21.5364	**21.8322**	21.1087	**0.7657**	0.7505	0.7461	0.7643	0.9748	0.9760	0.9739	**0.9763**
Img5	2	**175.242**	225.645	231.323	223.039	12.4456	11.9108	12.6701	**12.8356**	0.6563	0.6351	0.6576	**0.6830**	0.9646	0.9099	0.9613	**0.9658**
	3	205.294	223.118	220.808	**189.994**	13.6245	14.7228	15.4734	**15.5796**	0.7088	0.6913	**0.7326**	0.7141	**0.9578**	0.9657	0.9474	0.9506
	4	175.611	189.348	208.988	**167.488**	16.5684	16.4125	20.0959	**21.1263**	0.7286	0.6869	**0.7745**	0.7572	0.9641	0.9648	0.9620	**0.9692**
	5	163.711	198.021	**150.913**	172.097	17.750	18.2216	22.6659	**22.8863**	0.7464	0.7092	0.7886	**0.8277**	0.9706	0.9741	0.9772	**0.9802**
Img6	2	247.319	233.448	239.073	**232.398**	13.7352	14.2525	**14.4127**	14.2946	0.6704	0.6500	**0.6861**	0.6681	0.9436	0.9367	0.9621	**0.94803**
	3	224.428	235.524	227.418	**221.359**	16.2308	16.2704	17.2171	**18.3288**	0.7005	0.6772	**0.7212**	0.7161	0.9701	0.9656	0.9790	**0.98003**
	4	228.110	222.288	213.250	**197.527**	19.9904	18.8871	19.7375	**20.3376**	0.7274	0.7152	0.7426	**0.7464**	0.9825	0.9797	0.9847	**0.98443**
	5	**166.596**	168.018	173.173	188.119	20.9364	21.0873	21.3780	**21.7540**	0.7445	0.7456	0.7496	**0.7801**	0.9858	0.9853	**0.9887**	0.9884
Img7	2	219.250	220.827	234.351	**197.279**	15.1137	**18.7515**	18.0671	18.4292	0.6839	0.7035	0.6965	**0.7096**	0.9146	0.9237	0.9379	**0.9415**
	3	210.507	213.080	191.698	**183.035**	20.2030	17.8985	21.1190	**21.2213**	**0.7755**	0.6989	0.7290	0.7200	**0.9591**	0.8945	0.9526	0.9589
	4	**112.010**	227143	163.328	148.629	20.4950	20.3347	22.7456	**23.1965**	0.7654	0.7041	0.7469	**0.7731**	0.9627	0.9602	0.9563	**0.9680**
	5	131.061	201.400	**123.087**	126.241	23.0062	21.9584	23.0524	**23.8382**	**0.8116**	0.7197	0.8050	0.8041	0.9617	0.9651	0.9369	**0.9688**
Img8	2	**145.128**	244.11	244.005	224.643	**17.3964**	16.5393	16.4024	16.5517	**0.7213**	0.6558	0.6601	0.6806	0.7792	0.8071	0.8040	**0.8245**
	3	**152.507**	226.214	206.732	179.34	19.1499	18.3084	20.3699	**21.2332**	0.7624	0.6933	0.7557	**0.7739**	0.8436	0.8263	0.8623	**0.8704**
	4	218.206	223.677	181.118	**157.058**	19.7812	19.1445	22.6370	**23.0862**	0.7774	0.7077	0.8201	**0.8464**	**0.9114**	0.8387	0.8902	**0.9064**
	5	116.378	203.504	172.312	**117.823**	23.5620	21.2489	23.1626	**25.2347**	0.8702	0.7796	0.8343	**0.8712**	**0.9384**	0.8771	0.9005	0.9176
Img9	2	216.044	232.309	234.950	**215.202**	15.9663	17.1397	18.3113	**18.8499**	0.6649	0.6607	0.6869	**0.6962**	0.9278	0.9222	0.9501	**0.9583**
	3	198.979	238.836	229.886	**184.955**	17.9418	16.6143	18.9293	**21.2396**	0.6867	0.6671	0.7076	**0.7444**	0.9495	0.9232	0.9634	**0.9729**
	4	158.772	234.294	221.921	**157.763**	21.4965	18.4320	19.7220	**22.6152**	0.7671	0.6854	0.7271	**0.7872**	0.9739	0.9602	0.9671	**0.9785**
	5	**121.525**	204.242	163.736	134.367	22.886	20.7396	21.4092	**23.8510**	0.808	0.7397	0.7386	**0.8225**	0.9789	0.9712	0.9550	**0.9829**
Img10	2	249.483	**165.215**	243.583	170.210	15.3763	20.3859	19.4787	**20.8147**	0.7201	**0.7388**	0.7290	0.7381	0.8960	0.9308	**0.9489**	0.9470
	3	165.470	218.479	243.311	**136.404**	21.7684	18.7126	19.4453	**23.9158**	0.7348	07296	0.7346	**0.7617**	0.9500	0.9110	0.9509	**0.9581**
	4	172.672	198.025	217.935	**133.846**	24.6356	21.2853	19.3489	**24.9056**	**0.7969**	0.7360	0.7369	0.7672	0.9614	0.9417	0.9532	**0.9665**
	5	181.790	189.018	206.890	**116.888**	**25.8329**	22.3978	20.8161	25.2335	0.7139	0.7393	0.7484	**0.7814**	0.9729	0.9458	0.9451	**0.9743**

**Table 3:** COVIDOA-acquired segmented images at Th = 2, 3, 4, and 5 with proposed fitness function.

Original Image	Th2	Th3	Th4	Th5
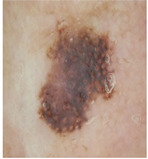 Img1	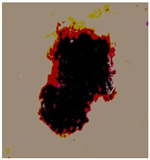	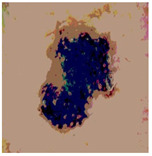	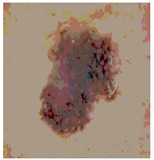	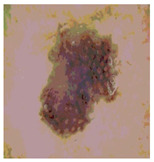
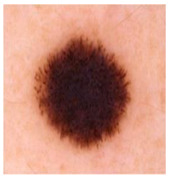 Img2	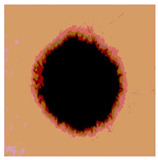	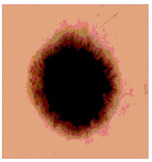	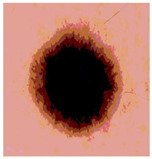	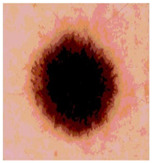
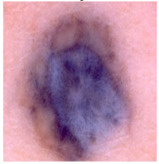 Img3	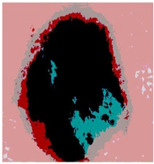	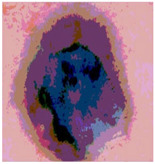	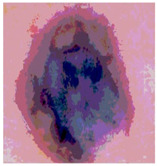	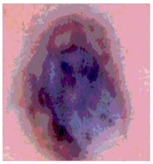
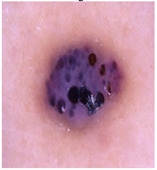 Img4	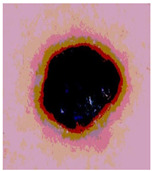	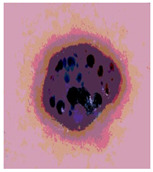	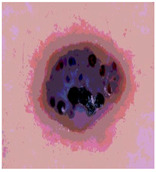	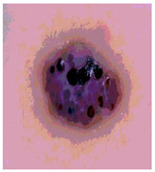
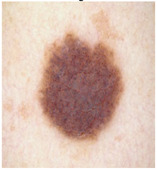 Img5	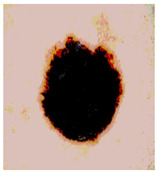	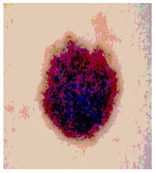	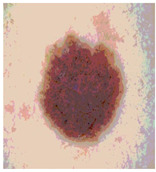	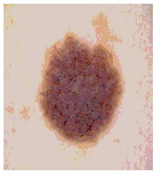
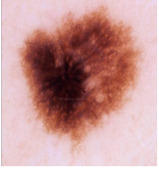 Img6	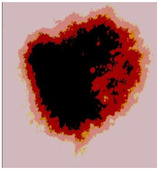	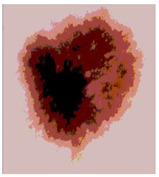	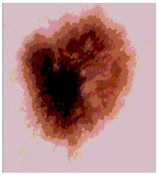	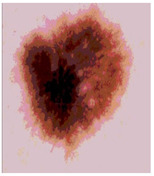
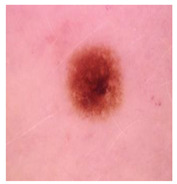 Img7	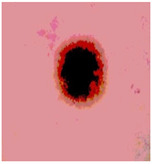	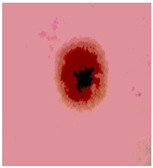	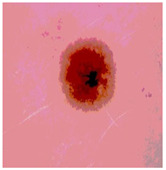	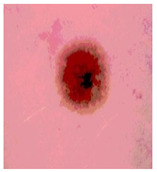
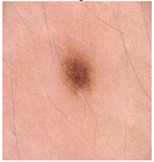 Img8	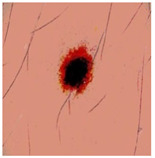	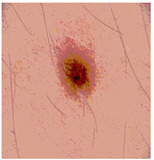	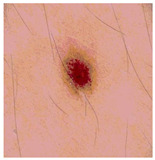	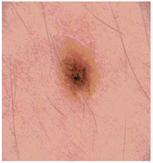
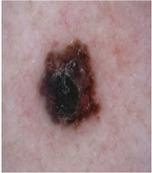 Img9	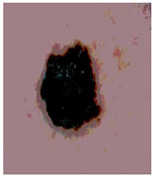	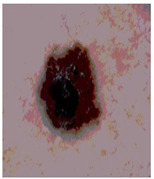	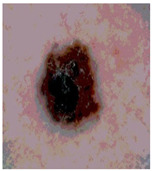	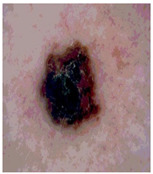
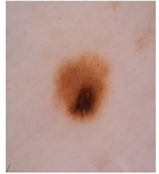 Img10	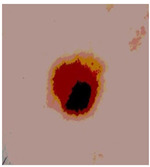	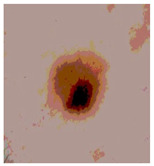	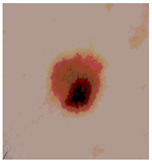	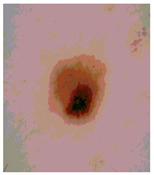

**Table 4:** COVIDOA example for segmented image (image 10) and RGB channel histograms computed at Th = 2, 3, 4, and 5.

Segmented Image	R	G	B
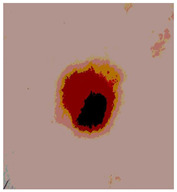	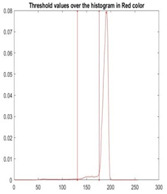	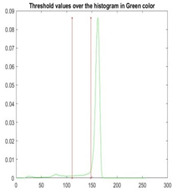	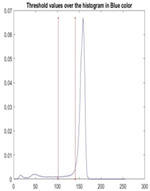
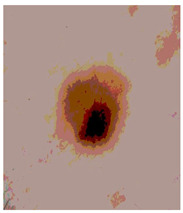	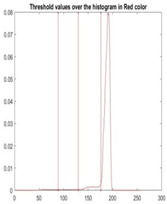	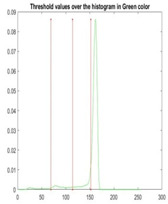	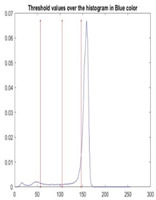
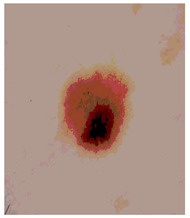	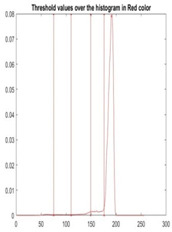	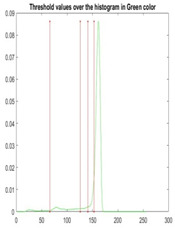	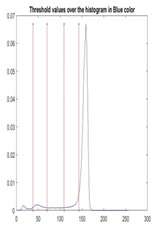
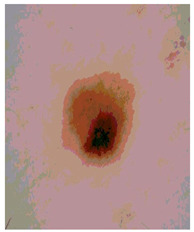	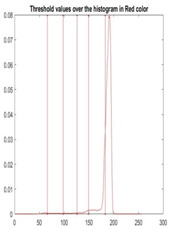	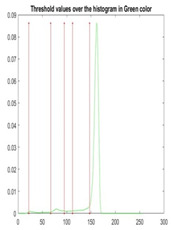	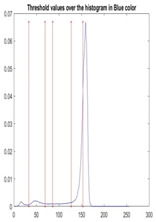

**Table 5:** Based on the average MSE values, a comparison of COVIDOA and the other chosen algorithms.

Image	Th	AOA	SCA	RSA	FPA	SOA	GTO	COVIDOA
Img1	2	247.6006	246.1469	247.8406	245.1597	246.5855	245.7669	**244.5473**
	3	225.2764	214.8453	205.2064	212.5436	223.9644	208.1518	**196.0748**
	4	221.2930	180.7156	203.4535	**141.8333**	205.8136	196.2350	196.1226
	5	241.1227	**144.8201**	214.7789	186.0322	198.2510	176.3300	162.9383
Img2	2	241.9144	241.8842	246.9748	**240.1664**	245.1630	242.1664	241.1411
	3	238.1451	237.3881	237.8582	239.1858	240.1110	239.1467	**222.2529**
	4	223.7759	233.3980	226.9811	228.1738	231.0375	220.5672	**206.8136**
	5	245.6072	216.9570	201.0338	205.5512	218.0571	209.0686	**186.7584**
Img3	2	236.8195	236.9835	**222.2556**	237.1442	237.0373	237.1505	228.0216
	3	216.8246	230.1775	209.0135	217.5782	217.9692	218.2340	**201.2815**
	4	207.5125	207.5971	205.1720	206.8667	207.8523	199.8962	**195.8324**
	5	223.7134	188.6496	190.1130	192.8761	193.0601	188.1727	**167.0796**
Img4	2	227.0835	230.8520	227.5441	230.5200	231.4647	230.5200	**202.0041**
	3	211.8023	220.8783	216.0401	224.9724	**195.7951**	221.5360	196.0211
	4	200.8875	223.9088	219.8616	206.1327	223.6879	216.2579	**187.2641**
	5	247.7828	201.5145	203.8915	206.1327	205.0691	207.6387	**171.1820**
Img5	2	**222.5750**	229.2426	238.2147	226.4986	227.2117	227.2265	223.0393
	3	209.4685	230.7308	**177.2353**	218.2365	226.2237	221.8623	**189.9940**
	4	215.9736	231.2013	222.5808	212.2887	218.8509	208.3670	**167.4879**
	5	250.5794	198.7918	182.3691	199.6067	197.7102	198.5641	**172.0972**
Img6	2	**232.2780**	233.2146	237.3292	235.6143	236.0539	235.6143	**232.2780**
	3	225.3670	**219.0977**	227.9831	226.8610	228.2006	228.8451	221.3586
	4	215.5507	226.2049	212.0640	217.9596	219.7537	210.2340	**197.5274**
	5	236.4972	226.2049	214.2859	202.7830	204.4251	199.7237	**188.1148**
Img7	2	222.5029	226.2049	248.0666	213.2920	219.6254	215.7291	**197.2790**
	3	212.5238	243.0814	239.0463	217.4714	222.2559	211.3120	**183.0348**
	4	189.3138	185.1541	204.6421	202.6493	206.4996	208.6859	**148.6285**
	5	205.7216	186.6668	231.7075	174.5481	219.3802	202.3541	**126.2406**
Img8	2	227.6083	251.3774	**219.0853**	232.5211	229.8166	229.8120	224.6433
	3	204.9689	221.0381	247.8927	217.7156	224.2208	216.4100	**179.3438**
	4	**153.7039**	229.0897	214.5952	212.1802	202.8934	208.5816	157.0576
	5	163.6398	198.8157	154.5615	207.6764	187.3008	197.6310	**117.8226**
Img9	2	222.6554	224.6326	247.6065	223.4470	224.5672	223.4491	**215.2015**
	3	204.5241	228.3561	237.1668	224.3819	214.2435	211.5460	**184.9549**
	4	189.5535	219.7749	180.9008	199.3643	219.6227	202.3142	**157.7634**
	5	246.8424	203.0891	187.9011	171.0936	201.3815	198.2314	**134.3671**
Img10	2	193.9262	219.9844	241.2282	**167.2653**	201.5983	191.9097	170.2101
	3	164.1696	251.9099	207.3865	183.4998	195.1959	193.4991	**136.4044**
	4	180.4451	249.6355	241.0450	177.0260	193.8249	177.1338	**133.8458**
	5	249.0814	219.0693	239.9401	218.1823	196.4945	152.3470	**116.8888**

**Table 6:** Based on the mean PSNR values, the COVIDOA and the other chosen algorithms are compared.

Image	Th	AOA	SCA	RSA	FPA	SOA	GTO	COVIDOA
Img1	2	12.6916	12.7582	12.7703	**12.8928**	12.5227	12.7635	12.8157
	3	15.479	15.6818	15.5330	15.3459	14.6225	15.5920	**16.2881**
	4	11.9246	**21.1715**	18.3366	20.4388	20.7199	19.6530	20.9091
	5	14.1807	20.0059	17.0006	21.8298	21.3421	**22.9359**	22.6851
Img2	2	15.2921	15.1074	13.4111	15.1709	14.5427	15.1708	**15.3446**
	3	16.6603	17.1524	17.1305	16.8841	16.7627	16.9005	**18.1292**
	4	18.1235	17.7695	**19.6477**	18.5581	18.3501	18.5623	19.4283
	5	13.5758	19.5727	18.8905	20.1187	19.6857	20.2480	**20.6270**
Img3	2	10.2965	10.3048	09.9944	10.3140	**10.9370**	10.3359	10.8181
	3	14.3920	13.1320	13.1290	14.6335	14.7912	14.9568	**16.6610**
	4	14.4746	16.8630	17.2929	19.0142	18.8696	19.0124	**19.3445**
	5	13.8634	17.3687	18.5697	20.7029	20.0024	**21.3329**	21.3038
Img4	2	14.0519	14.0302	**14.5456**	14.0325	14.0121	14.0325	14.4077
	3	18.1824	18.4383	17.1256	18.3327	18.1885	18.9654	**19.5479**
	4	18.8780	17.8194	17.3093	20.0309	18.4881	19.6989	**20.7163**
	5	10.9544	20.4764	18.0280	21.0277	20.6490	21.3457	**22.1088**
Img5	2	**12.8383**	12.7648	12.2583	12.8072	12.7768	12.7933	12.8356
	3	14.4969	14.1322	14.8782	15.6127	16.3258	**16.4677**	15.5796
	4	16.8143	14.6929	17.1395	20.4125	20.2356	21.0231	**21.1263**
	5	12.4279	20.8377	20.0745	21.7622	21.7227	21.9568	**22.8863**
Img6	2	14.1149	14.2493	12.4859	14.1924	14.2035	14.1924	**14.2946**
	3	17.6128	17.5604	15.9501	17.9081	17.8064	18.0124	**18.3288**
	4	19.2894	16.3362	18.0414	**20.4329**	19.2129	19.9823	20.3376
	5	13.6432	15.9573	17.5619	21.1660	21.3138	21.5138	**21.7540**
Img7	2	17.8573	9.0647	15.1141	18.1887	18.1174	18.2218	**18.4292**
	3	20.9240	15.7042	18.4234	20.9894	20.7422	20.4210	**21.2213**
	4	**23.5151**	20.3822	18.7204	21.9646	22.1361	22.0069	23.1965
	5	18.4159	21.7143	19.3929	22.9927	21.0572	22.8410	**23.8382**
Img8	2	16.3356	13.4726	14.8376	16.4363	**16.6193**	15.0121	16.5517
	3	20.7668	16.5391	16.4506	20.4295	20.1469	19.2310	**21.2332**
	4	22.6643	16.7415	17.0362	21.4753	21.7739	21.6554	**23.0862**
	5	22.2005	20.0964	23.0482	21.4175	22.7238	22.4057	**25.2347**
Img9	2	18.5839	18.5990	15.9292	**18.9314**	18.5884	18.6395	18.8499
	3	20.7089	16.8991	16.5721	19.5291	20.3260	21.0314	**21.2396**
	4	21.4919	16.5244	19.9028	21.0434	20.3115	21.9869	**22.6152**
	5	15.8094	15.2637	20.4012	21.9074	21.5602	22.3567	**23.8510**
Img10	2	20.5999	15.2959	18.0531	20.6405	20.4843	20.5994	**20.8147**
	3	23.2996	12.6441	19.8027	23.1252	**23.9542**	23.0295	23.9158
	4	22.7509	12.7175	18.8998	24.1067	23.5074	24.2329	**24.9056**
	5	15.2400	14.9047	18.5323	22.5299	23.9037	25.0195	**25.2335**

**Table 7:** Based on the mean FSIM values, a comparison of the COVIDOA and the other chosen algorithms.

Image	Th	AOA	SCA	RSA	FPA	SOA	GTO	COVIDOA
Img1	2	0.7378	**0.7389**	0.7359	0.7382	0.7230	0.7387	0.7387
	3	0.7586	0.7628	0.7356	0.7627	0.7227	**0.7667**	0.7654
	4	0.7506	0.7814	0.7649	0.7734	0.7707	0.7982	**0.7999**
	5	0.7365	0.7861	0.7556	0.8125	0.7608	0.8159	**0.8179**
Img2	2	0.6497	0.6501	**0.6586**	0.6503	0.6339	0.6503	0.6503
	3	**0.6696**	0.6666	0.6610	0.6685	0.6658	0.6683	0.6688
	4	0.6967	0.6892	0.6825	0.6892	0.6724	0.7002	**0.7018**
	5	0.6284	**0.7244**	0.6993	0.7168	0.7141	0.7205	**0.7244**
Img3	2	0.6684	0.6675	0.6716	**0.6690**	0.6524	0.6689	0.6612
	3	**0.6919**	0.6560	0.6823	0.6832	0.6906	0.6894	0.6870
	4	0.6873	0.6975	0.7124	0.7156	0.7134	0.7134	**0.7159**
	5	0.6762	0.7130	0.7365	**0.7457**	0.7194	0.7432	0.7308
Img4	2	0.6588	0.6618	0.6549	0.6623	0.6629	0.6623	**0.6717**
	3	0.7117	0.7060	0.7022	0.6990	0.6835	0.6936	**0.7169**
	4	0.7258	0.6906	0.7113	**0.7378**	0.7113	0.7151	0.7359
	5	0.6525	0.7331	0.7326	0.7312	0.7375	0.7521	**0.7643**
Img5	2	0.6807	0.6828	0.6745	**0.6830**	0.6827	0.6827	**0.6830**
	3	**0.7324**	0.6673	0.6870	0.7285	0.6942	0.7296	0.7141
	4	0.7255	0.7066	0.7574	**0.7766**	0.7721	0.7512	0.7572
	5	0.6582	0.7875	0.7831	0.8198	0.8117	0.8100	**0.8277**
Img6	2	0.6680	0.6690	0.6635	**0.6716**	0.6708	**0.6716**	0.6681
	3	0.7140	0.6868	0.7054	**0.7161**	0.7156	0.7013	**0.7161**
	4	0.7397	0.7382	**0.7676**	0.7423	0.7435	0.7469	0.7464
	5	0.6986	0.7468	0.7508	0.7617	0.7722	**0.7961**	**0.7801**
Img7	2	0.7007	0.6736	0.6888	0.7054	0.7032	0.7051	**0.7096**
	3	0.7142	0.6890	0.7097	0.7138	0.7120	0.7112	**0.7200**
	4	0.7320	0.7407	0.7005	0.7415	0.7250	0.7253	**0.7731**
	5	0.7088	0.7406	0.7087	0.7814	0.7403	0.7821	**0.8041**
Img8	2	0.6745	0.5882	0.6317	0.6726	**0.6881**	0.6780	0.6806
	3	0.7562	0.6674	0.6525	0.7459	0.7359	0.7532	**0.7739**
	4	0.8245	0.6624	0.7052	0.7736	0.7860	0.7801	**0.8464**
	5	0.8077	0.7626	0.8324	0.6869	0.8106	0.8061	**0.8712**
Img9	2	0.6925	0.6916	0.6740	0.6903	0.6869	0.6928	**0.6962**
	3	0.7320	0.6903	**0.7999**	0.7055	0.7234	0.7564	0.7444
	4	0.7516	0.6767	0.7241	0.7386	0.7219	0.7801	**0.7872**
	5	0.6561	0.6966	0.7493	0.7728	0.7545	0.8210	**0.8225**
Img10	2	0.7364	0.7254	0.7298	0.7374	0.7362	0.7369	**0.7381**
	3	0.7437	0.7188	0.7245	0.7454	0.7439	0.7437	**0.7617**
	4	**0.7687**	0.7198	0.7377	0.7507	0.7507	0.7522	0.7672
	5	0.7212	0.6696	0.7377	0.7490	**0.7844**	0.7785	0.7814

**Table 8:** Based on the mean NCC values, a comparison of the COVIDOA and the other chosen algorithms.

Image	Th	AOA	SCA	RSA	FPA	SOA	GTO	COVIDOA
Img1	2	0.9150	**0.91607**	0.9155	0.9142	0.9125	0.9160	0.9152
	3	0.9105	0.9197	0.9080	0.9231	0.9148	0.9232	**0.9238**
	4	0.3242	**0.9372**	0.9280	0.9271	0.8974	0.9312	**0.9372**
	5	0.9138	0.9420	0.8947	0.9404	0.9417	**0.9651**	0.9523
Img2	2	**0.9628**	0.9624	0.9504	0.9627	0.9497	**0.9628**	0.9627
	3	0.9778	0.9777	0.9785	0.9787	0.9719	0.9788	**0.9808**
	4	0.9812	0.9815	0.9794	0.9841	0.9778	0.9821	**0.9852**
	5	0.9483	0.9840	0.9746	0.9872	0.9700	**0.9881**	0.9879
Img3	2	0.9055	0.9060	0.9058	0.9063	0.9064	0.9063	**0.9146**
	3	0.9272	0.7407	**0.9359**	0.9276	0.9215	0.9332	0.9332
	4	0.9031	0.9299	0.9326	**0.9585**	0.9515	0.9573	0.9542
	5	0.9030	0.9163	0.9452	0.9668	0.9519	**0.9728**	0.9684
Img4	2	0.9162	0.9172	0.9103	0.9174	0.9176	0.9174	**0.9292**
	3	0.9550	0.9532	0.9499	0.9535	0.9509	0.9364	**0.9622**
	4	0.9579	0.9394	0.9201	0.9677	0.9629	0.9578	**0.9693**
	5	0.8117	0.9676	0.9527	0.9757	0.9715	0.6756	**0.9763**
Img5	2	0.9655	0.9641	0.9550	0.9648	0.9627	0.9646	**0.9658**
	3	0.9598	0.9351	0.9521	**0.9686**	0.9664	0.9684	0.9506
	4	0.9620	0.9117	0.9625	0.9656	0.9674	0.9675	**0.9692**
	5	0.9476	0.9710	0.9682	0.9715	0.9762	0.9754	**0.9802**
Img6	2	0.9459	0.9478	0.9269	0.9478	**0.9481**	0.9478	0.9480
	3	0.9773	0.9783	0.9612	0.9796	0.9797	0.9634	**0.9800**
	4	0.9820	0.9673	0.9808	**0.9850**	0.9819	0.9805	0.9844
	5	0.9499	0.9498	0.9739	0.9859	0.9854	**0.9900**	0.9884
Img7	2	0.9384	0.7331	0.9254	0.9407	0.9379	0.9408	**0.9415**
	3	**0.9589**	0.9295	0.9456	0.9576	0.9578	0.9521	**0.9589**
	4	0.9636	0.9318	0.9127	0.9629	0.9647	0.9643	**0.9680**
	5	0.9180	0.9506	0.9492	0.9657	0.9606	0.9621	**0.9688**
Img8	2	0.8210	0.7439	0.8228	0.8193	0.8220	0.8159	**0.8245**
	3	0.8635	0.8379	0.7984	0.8593	0.8572	0.8501	**0.8704**
	4	0.9032	0.7676	0.7643	0.8732	0.8812	0.8784	**0.9064**
	5	0.8846	0.8582	0.8829	0.8790	0.8930	0.8897	**0.9176**
Img9	2	0.9569	0.9574	0.9473	0.9550	0.9566	0.9574	**0.9583**
	3	0.9703	0.9285	0.9468	0.9659	0.9694	0.9713	**0.9729**
	4	0.9727	0.9261	0.9530	0.9665	0.9691	**0.9790**	**0.9785**
	5	0.9255	0.8852	0.9727	0.9705	0.9728	0.9799	**0.9830**
Img10	2	0.9402	0.8591	0.9199	0.9406	0.9405	0.9405	**0.9470**
	3	0.9579	0.8472	0.9350	0.9500	0.9642	**0.9648**	0.9582
	4	0.9552	0.8387	0.8822	0.9629	0.9679	**0.9694**	0.9665
	5	0.8850	0.8219	0.9426	**0.9677**	0.9420	0.9672	0.9543

## Data Availability

The data are available at https://www.kaggle.com/datasets/pardonndlovu/chestpelviscspinescans (accessed on 17 August 2023).

## Abbreviations

SC	Skin Cancer
UVR	Ultraviolet Radiation
MLT	Multilevel Thresholding
COVIDOA	Coronavirus Disease Optimization Algorithm
AOA	Arithmetic Optimization Algorithm
SCA	Sine Cosine Algorithm
RSA	Reptile Search Algorithm
FPA	Flower Pollination Algorithm
SOA	Seagull Optimization Algorithm
GTO	Gorilla Troops Optimizer
MSE	Mean Square Error
PSNR	Peak Signal-to-Noise Ratio
FSIM	Feature Similarity Index Metric
NCC	Normalized Correlation Coefficient
CAD	Computer-Aided Diagnosis
AI	Artificial Intelligence
PSO	Particle Swarm Optimization
WOA	Whale Optimization Algorithm
CSA	Cuckoo Search Algorithm
HHOA	Harris Hawks Optimization Algorithm
GWOA	Gray Wolf Optimization Algorithm
EOA	Equilibrium Optimization Algorithm
COA	Chimp Optimization Algorithm
MRFOA	Manta Ray Foraging Optimization Algorithm
SMA	Slime Mould Algorithm
MPA	Marine Predators Algorithm
BWOA	Black Widow Optimization Algorithm
MGWO	Multistage Grey Wolf Optimizer
VCS	Virus Colony Search
SSA	Salp Swarm Algorithm
FA	Firefly Algorithm
OBL	Opposition-Based Learning
ABC	Artificial Bee Colony
KHO	Krill Herd Optimization
DBN	Deep Belief Network
MEFOA	Modified Electromagnetic Field Optimization Algorithm
MAFBUZO	Multi-Agent Fuzzy Buzzard Algorithm
ISIC	International Skin Imaging Collaboration
